# Enhanced Corrosion Resistance and Mechanical Durability of the Composite PLGA/CaP/Ti Scaffolds for Orthopedic Implants

**DOI:** 10.3390/polym16060826

**Published:** 2024-03-15

**Authors:** Konstantin A. Prosolov, Ekaterina G. Komarova, Ekaterina A. Kazantseva, Nikita A. Luginin, Alexander D. Kashin, Pavel V. Uvarkin, Yurii P. Sharkeev

**Affiliations:** Laboratory of Physics of Nanostructured Biocomposites, Institute of Strength Physics and Materials Science, Siberian Branch of Russian Academy of Sciences, 634055 Tomsk, Russia; konstprosolov@gmail.com (K.A.P.); kazantseva.ea@ispms.ru (E.A.K.); nikishek90@gmail.com (N.A.L.); kash@ispms.ru (A.D.K.); uvarkin@ispms.ru (P.V.U.); sharkeev@ispms.ru (Y.P.S.)

**Keywords:** ultrasonic-assisted micro-arc oxidation, poly(lactic-co-glycolic acid) (PLGA), calcium phosphate coating, mechanical durability, corrosion resistance

## Abstract

In addressing the challenge of enhancing orthopedic implants, 3D porous calcium phosphate (CaP) coatings on titanium (Ti) substrates modified with poly(lactic-co-glycolic acid) (PLGA) were proposed. CaP coatings on Ti were deposited using the ultrasonic-assisted micro-arc oxidation (UMAO) method, followed by modification with PLGA through a dip coating process at concentrations of 5%, 8%, and 10%. The addition of PLGA significantly improved adhesive–cohesive strength according to the scratch test, while PLGA to CaP adhesion was found to be not less than 8.1 ± 2.2 MPa according to the peel test. Tensile testing showed a typical fracture of CaP coatings and mechanisms of brittle fracture. Corrosion resistance, assessed via gravimetric and electrochemical methods in 0.9% NaCl and PBS solutions, revealed PLGA’s substantial reduction in corrosion rates, with the corrosion current decreasing by two orders of magnitude even for the 5% PLGA/CaP/Ti sample. Also, the PLGA layer significantly enhanced the impedance modulus by two orders of magnitude, indicating a robust barrier against corrosion at all PLGA concentrations. Higher PLGA concentrations offered even greater corrosion resistance and improved mechanical properties. This research underscores the potential of using CaP- and PLGA-modified coatings to extend the life and functionality of orthopedic implants, addressing a significant challenge in biomedical engineering.

## 1. Introduction

The quest for effective drug delivery systems, particularly in the realm of bone treatment, remains a formidable challenge in modern medicine [1,2]. Central to this challenge is the development of efficient carriers that can precisely target and release therapeutic agents to diseased or damaged bone tissues. In this context, the surface engineering of metallic implants has emerged as a crucial strategy, increasing the strength and biocompatibility of metals to create advanced therapeutic devices [3]. Among various techniques, the electrochemical family of methods for modifying the surfaces of metallic implants stands out as particularly promising [4]. These methods not only enhance the mechanical properties of the implants but also offer a platform for drug incorporation and controlled release.

The micro-arc oxidation (MAO) method is a unique plasma-chemical and electrochemical process. MAO enables the formation of robust and biocompatible oxide layers on metal surfaces, particularly on widely used materials like Ti. In particular, various calcium phosphate-based coatings are the most promising due to the presence of vital elements such as Ca and P [5,6]. These oxide layers, characterized by their porous structures, are ideal for loading with therapeutic agents. By adjusting the parameters of the MAO process, it is possible to control the porosity, thickness, and composition of the coatings, thereby tailoring the implant’s surface for specific drug delivery applications [7]. In recent years, the field of biomedical implants improved and advanced due to the improved MAO method, which integrates ultrasonic (US) vibrations in the electrolyte during the coating process. This innovative approach, known as ultrasonic-assisted micro-arc oxidation (UMAO) [8,9], has garnered extensive research interest due to its potential in improving the properties of metallic implants, especially for applications in bone treatment. The UMAO method represents a synergistic combination of plasma-chemical and electrochemical reactions with the mechanical effects of ultrasonic vibrations. This integration has been shown to refine the microstructure of the coatings formed on Ti alloys. The enhanced microstructure attributed to UMAO offers improved mechanical properties, increased corrosion resistance, and potentially better bioactivity compared to conventional MAO coatings [10,11]. Furthermore, the incorporation of ultrasonic vibrations is found to influence the porosity and surface topography of the coatings, which are aspects that are crucial for drug encapsulation and controlled release in drug delivery systems [12]. This makes UMAO a promising technique for developing multifunctional implants that not only provide structural support but also actively participate in the therapeutic process [13]. The exploration of UMAO in recent studies proved the viability of this approach to manufacture new drug delivery systems [7].

The integration of drug delivery functionalities into metallic implants through surface engineering addresses multiple challenges in bone treatment [14]. It offers the potential for localized therapy, reducing systemic side effects and improving the efficacy of treatments. Furthermore, the ability to control drug release kinetics is a significant advantage, ensuring sustained therapeutic effects over prolonged periods, which is particularly beneficial in the context of bone healing and regeneration. However, controlled release is hard to achieve without the additional modification of UMAO coatings, CaP in particular. Therefore, additional modification by the addition of new encapsulation layers [15,16], functional groups [17,18] or the structurization of the surface on a nanoscale level [19] is usually employed.

Here, we proposed to use biodegradable poly(lactic-co-glycolic acid) (PLGA) at different concentrations to manipulate the drug delivery rate [7]. PLGA is known for its hydrolytic degradation, where the polymer chain is broken down into lactic acid and glycolic acid [20]. The rate of degradation of PLGA is influenced by factors like copolymer ratio, crystallinity, and molecular weight. Generally, PLGA degrades over a period of weeks to months, which can be tailored to match the desired rate of drug release or tissue regeneration. The degradation rate is crucial in medical applications, as it impacts the release kinetics of encapsulated drugs and the material’s structural integrity over time. In contrast, CaP coatings applied via MAO are more stable and degrade much slower than PLGA [21]. Even though these coatings are usually in an X-ray amorphous state and believed to be much more resorbable than crystalline hydroxyapatite, they are still quite stable in physiological solution and degrade through a process of dissolution and cellular activity over a prolonged period of time [22,23]. Biodegradation rates of CaP coatings, deposited using hybrid UMAO technique (UMAOH) (where H stands for hybrid and indicates that the US vibrations are switched off during the last minutes of deposition), which are later to be encapsulated by PLGA are important to consider and thoroughly research.

Furthermore, the wear resistance of MAO-deposited CaP coatings and those modified by PLGA is of vital importance, since this composite is designed to be used for bone implants with drug eluting capabilities. Frequently, MAO is used to form barrier Ti oxide layers that could withstand high-friction loads [24]. In other work, to further improve cell response, Ti oxide was enriched by Ca and P [25]. It is known that benefits from the dense and hard surface layer formed during the MAO process result in their higher toughness and wear resistance. On the other hand, amorphous CaP coatings are more bioactive due to the more rapid resorption rate [26]. Therefore, it is crucial to study its mechanical properties, especially in combination with the PLGA encapsulating layer. To our knowledge, the mechanical properties of CaP coatings modified by PLGA layers as well as the corrosion behavior of such composites are not well researched.

The integration of PLGA, a biodegradable polymer, with MAO CaP coatings could potentially offer enhanced mechanical properties, biocompatibility, and controlled drug release capabilities. Such coatings would be particularly beneficial in biomedical applications, such as orthopedic and dental implants. The aim of this research lies in exploration of PLGA/CaP/Ti scaffolds’ wear resistance, adhesion to the substrate, and tensile strength as well as their biodegradation rate and electrochemical parameters.

## 2. Materials and Methods

### 2.1. Sample Preparation

Commercially pure Ti (ASTM Grade 2) with a thickness of 1 mm and width and length of 10 mm (VSMPO-AVISMA Corp., Verkhnaya Salda, Russia) was used as the substrate for deposition. Before the deposition step, Ti samples were mechanically grinded and polished using silicon carbide paper with a grade ranging from P600 to P2000. A US cleaning (Elmasonic S, Elma Schmidbauer GmbH, Singen, Germany) in distilled water and ethanol for 10 min was performed. After that, the samples were dried in the air.

At the first step, the samples were functionalized by CaP coatings using the hybrid UMAO method. The UMAO method was realized by the Microarc-3.0 installation (ISPMS SB RAS, Tomsk, Russia) consisting of a pulsed DC power supply, a sample holder (anode), a titanium electrolytic bath (cathode), US devices, and a PC. The electrical parameters were the following: anodic static voltage of 200 V, pulse frequency of 50 Hz, pulse duration of 100 µs, time of 10 min, sinewave US with frequency of 35 kHz and capacity of 100 W. These conditions where applied for the initial 8 min and were switched off for the final 2 min, as in the previous works [7,27]. The electrolyte contained the following: nanosized hydroxyapatite (HA) (Ca_10_(PO_4_)_6_(OH)_2_), 5 wt.%; CaCO_3_, 7 wt.%; H_3_PO_4_, 27 wt.%; and distilled water as a balance. At the second step, the coated samples were immersed 10 times in the 5, 8, and 10 wt.% poly(D,L-lactide-co-glycolide) (PLGA) 50/50 (Mw = 56 kDa) (Novoсhem Corp., Tomsk, Russia) dissolved in trichloromethane [27]. Then, the samples were dried in the vacuum drying oven for 12 h at 40 °C.

### 2.2. Scanning Electron Microscopy

Coating structure and morphology were analyzed by scanning electron microscopy (SEM, LEO EVO 50, Carl Zeiss, Oberkochen, Stuttgart, Germany). The thickness was measured using SEM images of the coating cross-sections according to the ASTM E1382-9 standard [28]. Experimental equipment was provided by the “Nanotech” Common Center for Collective Use (ISPMS SB RAS, Tomsk, Russia).

### 2.3. Peel Adhesion and Scratch Tests

The adhesion strength of the composite layers, comprising CaP to the Ti substrate, was evaluated using the uniform peel adhesion method, as per ISO 13779-4-2018 and ASTM F1147 standards [29,30]. This assessment was conducted on a Walter+Bai AG LFM (Löhningen, Switzerland) testing machine.

**Figure 1:**
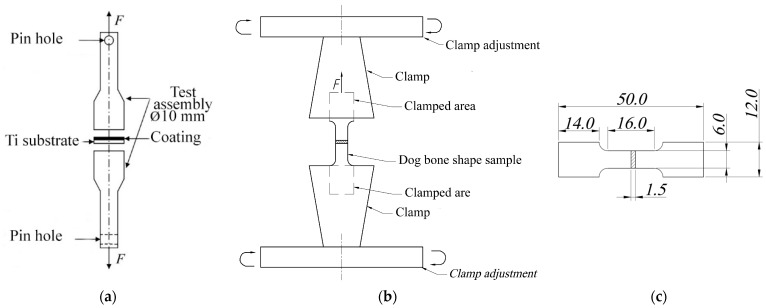
Schematic illustration of a peel adhesion (**a**) and tensile test (**b**) with a corresponding dimension (in mm) of a dog bone sample plate (**c**).

For the test, metal cylinders were securely glued to both sides of the coated samples including CaP/Ti and PLGA/CaP/Ti with different concentrations of polymer (Figure 1a). These samples were then firmly clamped with a vice on the testing machine. The adhesion strength was measured by stretching the samples at room temperature, with the machine’s clamps moving at a constant displacement speed of 0.1 mm/min. Adhesion strength was calculated as the critical stress (*σ_f_*) at which the coating was separated from the substrate, using the following formula:

(1) $$\sigma_{f}=\frac{P}{\pi \cdot a^{2}}$$

where *P* is the critical load at which the cylinder was separated from the sample, and *a* is the radius of the surface of the metal cylinder in contact with the sample (5 mm).

An orthogonal method used for determining the adhesion strength of the composite layers PLGA/CaP to the Ti substrate was sclerometry (scratch testing). Measurements were carried out using the Revetest CSM Instruments SA (Anton Paar, Graz, Austria) scratch tester equipped with a Rockwell diamond indenter (radius of 200 μm). This method involved applying a linearly increasing load up to 40 N (scratch length of 5 mm, indenter movement speed of 2.5 mm/min). Prior to that, using a surface profiler KLA-Tencor Alpha-Step IQ (San Jose, CA, USA), a surface roughness (*Ra*) was measured for all test samples.

The mechanism of adhesive failure of the composite coating was evaluated using optical images of the scratches. Additionally, the study included analysis of the coefficient of friction (*µ*) as a ratio of friction force to load, the patterns of acoustic emission (*AE*) generated during the coating’s destruction, and the dependency of the indenter penetration depth (*P_d_*) on the scratch length (*d*) or the magnitude of the load (*F_n_*). This comprehensive approach provided a detailed understanding of the adhesion properties and the mechanical behavior of the composite layers on the Ti substrate and allowed us to compare two widely used approaches for coating adhesion determination.

### 2.4. Tensile Test

For investigating the deformation behavior of CaP/Ti, tensile experiments (Figure 1b) were conducted on samples shaped like dog bone made of pure Ti, with dimensions according to GOST 11701-84 standard [31] (Figure 1c). The tests were carried out on two groups of samples: pure Ti (control group); and CaP/Ti composite. The tensile tests on these samples were performed using a Walter+Bai AG LFM (Löhningen, Switzerland) universal testing machine, with a clamp displacement speed of 0.1 mm/min and a continuously increasing load. The stretching process was recorded using a digital video camera. During the tensile tests, stress–strain diagrams were obtained for both pure Ti and the CaP/Ti composite. 

To ascertain the contribution of the CaP coating to the composite’s deformation behavior, the samples were stretched in two modes: (1) stretching the entire CaP/Ti system, with the CaP coating inserted in the clamps, and (2) stretching only the Ti component, without the CaP coating in the clamps. From the stress–strain diagrams, the conventional yield strength σ_0.2_ and the elastic limit σ_0.02_ (in the absence of a yield plateau) were determined by drawing lines from points corresponding to 0.2% and 0.02% strains, parallel to the line representing the elastic part of the diagram, as per GOST 1497-84 [32]. The Young’s modulus of the samples was calculated as the tangent of the angle of inclination of the elastic portion of the stress–strain diagram.

### 2.5. Immersion Test

The study of the biodegradation kinetics of all sample groups, namely CaP/Ti and PLGA/CaP/Ti at different polymer concentrations, was conducted using a gravimetric method in a 0.9 wt.% NaCl solution (0.9% NaCl) and phosphate-buffered saline (PBS) at 37 °C for 21 days. The test procedure was performed in accordance with the ASTM G31-21 standard [33]. The change in the mass of the samples was monitored by weighing them at 2 day intervals. The relative mass loss was calculated according to the following formula:

(2) $$\frac{∆m}{m_{0}}=\frac{m_{0}-m}{m_{0}}\times 100\%$$

where *m*_0_ is the initial mass of the sample before testing and *m* is the mass of the sample measured at a certain point in time.

### 2.6. Electrochemical Impedance Spectroscopy

The study of the electrochemical properties was conducted using potentiodynamic polarization and electrochemical impedance spectroscopy (EIS) methods. Measurements were carried out on a P-40X potentiostat-galvanostat with an FRA-24 frequency analysis module in a three-electrode E-7SF cell (Electrochemical Instruments Corp., Chernogolovka, Russia) at room temperature. The electrolytes used were 0.9% NaCl and PBS. The setup included a graphite counter electrode and a 4.2 M silver chloride reference electrode.

Potentiodynamic curves were recorded with a sweep rate of 2 mV/s over a range of ±500 mV from the open circuit potential for each sample. To stabilize the electrochemical processes at the electrolyte/material interface, each sample was pre-soaked in the solution within the cell for 60 min before the experiment commenced. Polarization curves were plotted to determine the corrosion characteristics, following the Tafel equation with a correlation coefficient of at least 0.998 and an acceptable potential error of no more than 1 mV, using software ES8 v. 4.1932. The polarization resistance (R_p_) calculation was conducted using the Stern–Geary equation. EIS was performed in a potentiostatic mode at the open circuit potential, with an amplitude sweep of 50 mV over a frequency range from 1 × 10^5^ to 5 × 10^−2^ Hz. Impedance measurement results were approximated using software ZView v. 3.2b and a modified nonlinear least squares method.

### 2.7. Statistical Analysis

The normality of the experimental data distribution was defined by the Kolmogorov–Smirnov test with Lilliefors correction. When describing the normal distribution, the mean (X) ± standard deviation (SD) was used to determine the central tendency. 

## 3. Results and Discussion

### 3.1. Morphology of PLGA/CaP/Ti Composite

**Figure 2:**
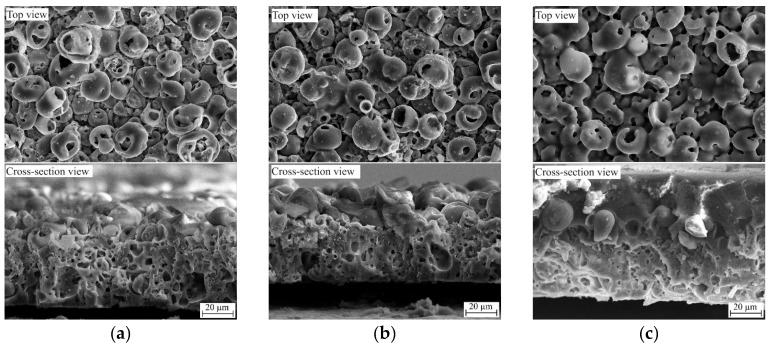
SEM top view and cross-section images of CaP/Ti (**a**), 5% PLGA/CaP/Ti (**b**), and 10% PLGA/CaP/Ti (**c**) scaffolds.

The cross-sectional morphology of the CaP and 5% PLGA/CaP/Ti samples were observed to be similar, both exhibiting uniform coatings with thicknesses ranging from 50 to 55 μm. Notably, these coatings are characterized by volumetric 3D porosity, as evident in Figure 2a. The surface morphology is distinguished by spherical structural elements (spheres) that possess pores both within their volume and interspersed between the spheres, as shown in Figure 2a. A detailed surface analysis and elemental composition evaluation of such composites have been reported in our previous works [27,34].

In contrast, samples with higher PLGA concentrations (8–10% PLGA/CaP/Ti) exhibited a two-layered structure. These coatings were heterogeneous in thickness, varying between 60 and 80 μm, and composed of a lower porous CaP layer topped by a denser upper polymer layer, depicted in Figure 2c. The introduction of PLGA significantly alters the coating’s morphology; the PLGA layer is uniformly distributed across the coating’s surface, enveloping the structural elements. This results in the complete overgrowth of both surface elements and pores, as demonstrated in Figure 2c. However, according to the surface roughness analysis, the *Ra* values measured were as follows: 3.3 ± 0.4 µm for CaP/Ti, 3.1 ± 0.4 µm for 5% PLGA/CaP/Ti, 3.0 ± 0.3 µm for 8% PLGA/CaP/Ti, and 2.4 ± 0.5 µm for 10% PLGA/CaP/Ti [27]. Therefore, in this study, we assume that the surface roughness of the produced samples does not significantly influence the adhesion values in both types of tests.

It is worth mentioning that the mechanical properties of porous materials, such as compressive strength and elasticity, are crucial, especially in load-bearing applications like orthopedic implants. The encapsulation of a CaP coating in a thick PLGA layer could significantly impact mechanical and corrosion properties. In the case of biodegradable materials like PLGA/CaP/Ti, the rate of degradation is significantly influenced by porosity. A well-designed porous structure can result in a tailored biodegradation rate that is congruent with tissue regeneration, ultimately leading to the replacement of the scaffold with natural tissue.

### 3.2. Adhesion Strength between PLGA/CaP/Ti Layers and Ti Substrate

**Figure 3:**
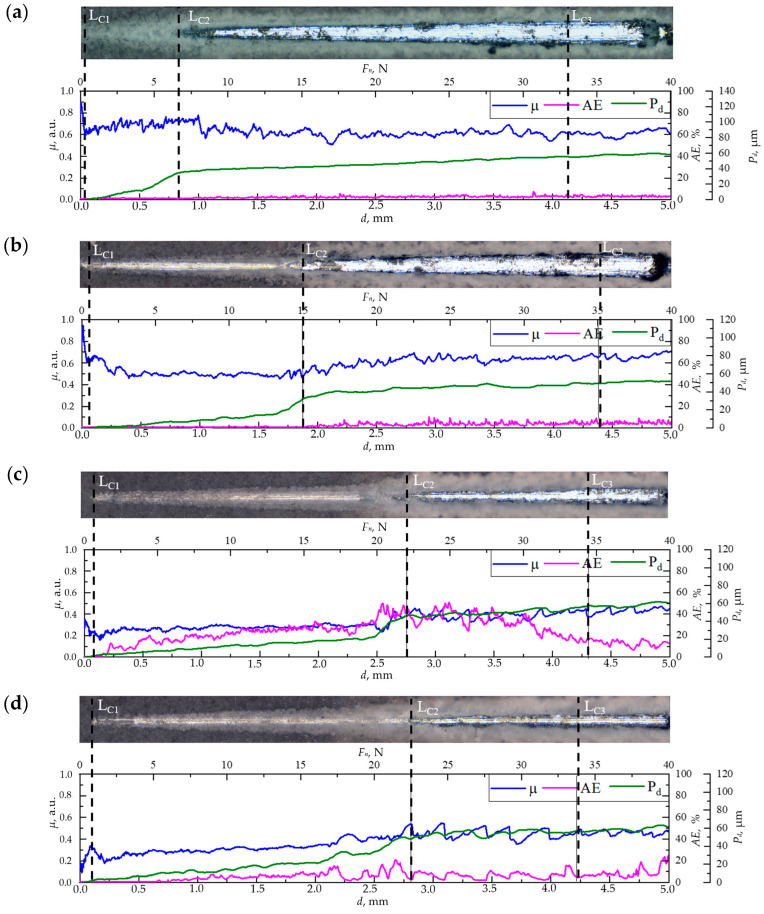
Optical images of scratches on the surface of CaP/Ti (**a**), 5% PLGA/CaP/Ti (**b**), 8% PLGA/CaP/Ti (**c**), and 10% PLGA/CaP/Ti (**d**) scaffolds after scratch testing, as well as the dependence of the friction coefficient (*µ*), acoustic emission (*AE*), and indenter penetration depth (*P_d_*) on scratch length or load.

In mechanical evaluations using scratch testing, distinct differences in the destruction mechanisms were observed across the CaP/Ti sample and the PLGA/CaP/Ti samples with different polymer concentrations. The pristine CaP coating exhibited early failure at low loads, starting at a mere 0.5 N (CL1) and extending to 7.1 N (CL2). This was evidenced by a superficial indenter mark seen in optical images, minimal acoustic emission (*AE*) signals below 1.7%, and a modest indenter depth of no more than 35.4 μm, as shown in Figure 3a.

When subjected to loads between 7.1 and 33.3 N (CL2 to CL3), the coating’s destruction transitioned to an adhesive–cohesive mechanism. This was indicated by the exposure of the bare Ti substrate within the scratch, as seen in optical images, alongside remnants of the intact coating at the scratch’s edges. During this phase, *AE* signals rose to 7%, and the indenter penetrated deeper, reaching up to 54.7 µm. Beyond the critical load of 33.3 N, the coating underwent complete delamination in an adhesive manner, indicating a total failure of the interface between the coating and substrate.

The incorporation of PLGA into the CaP coatings markedly improved their adhesive strength, as evidenced by increased critical loads (CL1, CL2, CL3) in comparison to the CaP/Ti (Figure 3b–d). Notably, the greatest enhancement was observed at CL2, the onset of adhesive–cohesive destruction. Increasing the PLGA concentration to 5% and 8–10% elevated the critical load to 15.0 and 22.9 N, respectively. In the range between CL1 and CL2, the indenter moved linearly and penetrated to depths of 15 and 21 μm for the 5% and 8–10% PLGA/CaP/Ti samples, respectively. This, alongside optical imaging, suggests that the PLGA underwent plastic deformation, as reported elsewhere [35], effectively preventing the brittle failure of the CaP coating.

Further scratching near the CL2 threshold revealed a sharp increase in indenter penetration depth to 33 and 50 μm for the 5% and 8–10% PLGA/CaP/Ti samples, respectively. This likely indicates the indenter’s contact with the CaP layer and the onset of coating degradation. The plastically deformable nature of PLGA acts as a deflector for crack tips formed during the indenter’s fracturing process. As the PLGA thickness increases, these cracks encounter more resistance, necessitating higher critical shear stress for propagation through the coating’s entirety. This phenomenon is consistent with the observed trend of increasing friction coefficient and high *AE* values in the 8–10% PLGA/CaP/Ti samples.

These results align with findings from other studies. For instance, research on a multilayer drug delivery system comprising a Ti substrate, gentamicin, and a thin PLGA surface layer showed similar enhancements in mechanical stability [36]. Additionally, a study on HA/chitosan-containing MAO coatings modified with PLA polymer demonstrated improved surface densification and crack prevention, enhancing the corrosion resistance of the magnesium substrate [37].

**Figure 4:**
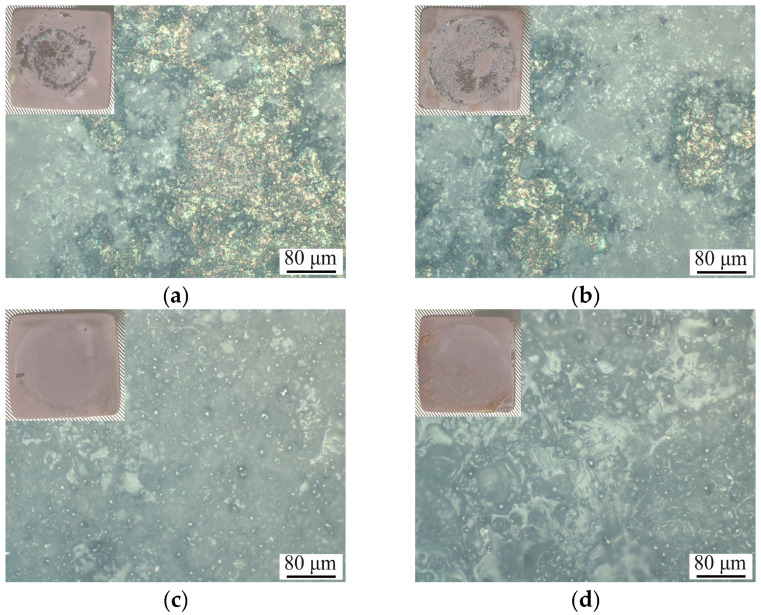
Optical images and corresponding photograph in the inset of CaP/Ti (**a**), 5% PLGA/CaP/Ti (**b**), 8% PLGA/CaP/Ti (**c**) and 10% PLGA/CaP/Ti (**d**) coatings after peel adhesion test.

Tension testing, performed according to standards [29,30], provided insightful data on the adhesive–cohesive strength of the composite layers to the Ti substrate. Intriguingly, incorporating a polymer into the CaP coating and increasing its concentration resulted in a decrease in adhesive–cohesive strength from 20.1 to 8.1 MPa (Table 1). The analysis of the samples post-testing, through photographs and optical imaging, revealed a mixed mode of failure. For CaP and 5% PLGA/CaP/Ti samples, the surfaces displayed both bare Ti substrate and residual CaP coating, indicating an adhesion–cohesion type of failure, as seen in Figure 4. However, in the 8–10% PLGA/CaP/Ti samples, no Ti substrate exposure was observed post-tearing. Instead, the surface was covered uniformly with the CaP coating and fragmented polymer areas, suggesting a cohesive failure at the CaP/PLGA interface. In other words, in cases of 8% PLGA/CaP/Ti and 10% PLGA/CaP/Ti, we were able to determine the value of PLGA to CaP adhesion which, to our knowledge, have not been reported previously, although the importance of this parameter is obvious and has been already indicated by other authors [38].

The variances in destruction mechanisms and adhesive strengths across the sample groups are attributed to their structural nuances, as discussed earlier. CaP coatings, known for their high 3D porosity and roughness (*Ra* = 3–4 μm), maintain a consistent thickness of 50–55 μm. The application of a 5% PLGA layer does not significantly alter this morphology. Consequently, such coatings tend to fail at the interface with the substrate as well as internally, along the boundaries of pores, cracks, and interlayer junctions.

Conversely, the treatment of CaP/Ti with 8–10% PLGA leads to the formation of a dense polymer layer, up to 30 µm thick, effectively sealing the surface layer pores. This barrier impedes the penetration of adhesives into the coating’s pores, redirecting the rupture to occur predominantly along the PLGA/CaP boundary, where there is an absence of chemical bonding. This observation is in stark contrast to findings by other researchers [39], who noted that PLA polymer deposition on anodic MgO coatings significantly enhanced mechanical adhesion. In their study, the PLA acted as a physical barrier, improving the bond between the coating and the substrate and restricting liquid penetration, including adhesives. This comparison highlights the complexity in designing composite coatings for biomedical applications. The balance between mechanical integrity and structural features is delicate; while increased polymer concentration can enhance certain aspects like cohesive strength, it may concurrently weaken others, such as adhesive properties to the substrate or previous layer, being CaP, in our case.

To conclude this section, as reported in ref. [40], no single adhesion strength testing technique is universally accepted or applicable in all coating–substrate situations. An ideal adhesion strength testing method would measure only the coating adhesion; however, all known testing methods are affected by other properties of the coating and substrate system. Even though a standardized method of CaP coatings adhesion evaluation is advised by the ISO and ASTM test procedures [29,30] requires peel testing, it does not always yield correct results, especially in case of multilayered coating systems, as reported in this article. Orthogonal methods of coating adhesion evaluation should be used. The advantages of scratch testing in this regard include the following: (1) that it simulates the usage stress conditions of orthopedic implants more closely than tensile adhesion strength testing techniques; and (2) that it can be used to measure the adhesion strength of thin coatings without the risk of bonding agents penetrating the coating.

### 3.3. Tensile Stress–Strain Behavior of CaP/Ti Composite

In the development of multilevel drug carriers, understanding their deformation behavior under tensile stress, which can reach up to 20 MPa in bone tissue–implant scenarios [41,42], is crucial. Our tensile tests produced stress–strain diagrams for both pure Ti and the CaP/Ti composite. To ascertain the specific influence of the CaP coating on the composite’s deformation behavior, we employed a dual approach: stretching the entire CaP/Ti system with the coating secured within the clamps and separately testing the Ti substrate alone, with the coating outside the clamps.

**Figure 5:**
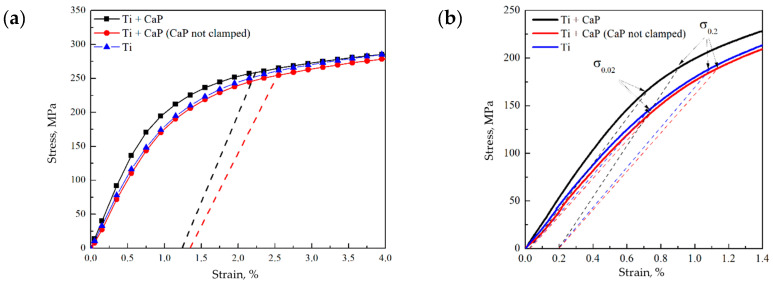
Stress–strain diagrams of the Ti and CaP/Ti samples: (**a**) the dotted lines indicate the stresses and residual strains at which cracks began to form in the CaP coating on the samples; (**b**) elastic region, where the dotted lines indicate the conditional elastic limit σ_0.02_ and yield strength σ_0.2_ of the samples.

The tensile strain–stress test results revealed a distinct difference in deformation behaviors. When the CaP coating was clamped, the CaP/Ti composite exhibited enhanced mechanical properties compared to both the uncoated Ti substrate and the coated substrate not secured in the clamps (Figure 5). Specifically, the presence of the CaP coating increased the elastic limit from 144.5 to 165.5 MPa and elevated the Young’s modulus from 22.1 to 26.2 GPa relative to the uncoated Ti substrate (Figure 5b). This suggests an effective stress redistribution between the Ti substrate and the CaP coating, enhancing the composite’s performance in the elastic region. Conversely, the mechanical properties of the composite with the unsecured coating were marginally reduced compared to the control. This reduction could be attributed to residual compressive stresses within the coating at its interface with the substrate. Supporting this, similar enhancements in mechanical characteristics were reported in an Al alloy with an MAO coating composed of two allotropic modifications of the Al_2_O_3_ crystalline phase [43].

It is noteworthy that pure Ti typically exhibits a high elastic modulus (around 120 GPa) [44]. However, our results showed that the Young’s modulus of Ti samples, both with and without a CaP coating, did not exceed 27 GPa. This discrepancy likely stems from the nuances of static testing. When Ti undergoes significant deformation at low loads, delineating the elastic deformation region from the transition to plastic deformation becomes challenging, leading to an ostensibly lower Young’s modulus. Crucially, for all samples, the conditional yield strength σ_0.2_ remained consistent, ranging between 187–200 MPa. This parameter is pivotal as it demarcates the end of elastic deformation and the onset of plastic deformation, indicating the robustness of the materials under mechanical stress.

Continuing from the robustness indicated by the σ_0.2_ values, the incorporation of uniaxial tension testing via in situ imaging has been pivotal in elucidating the failure mechanisms of the CaP coatings. High-speed video recording during these tensile tests allowed for the precise observation of the deformation levels and the corresponding stress points where crack nucleation in the CaP coatings commenced.

Interestingly, the CaP coating that was secured within the clamps began to collapse at a stress level of 258 MPa, while the coating outside the clamps started to fail at a slightly lower stress of 255 MPa (Figure 5a). These failure stresses are notably higher than the calculated yield strength σ_0.2_ of the composite samples (Figure 5b). It indicates that the coatings possess a considerable resistance to initial deformation. However, the residual deformation at which the CaP coating began to fail was limited to only about 1.3%, pointing toward a predominantly brittle fracture behavior.

**Figure 6:**
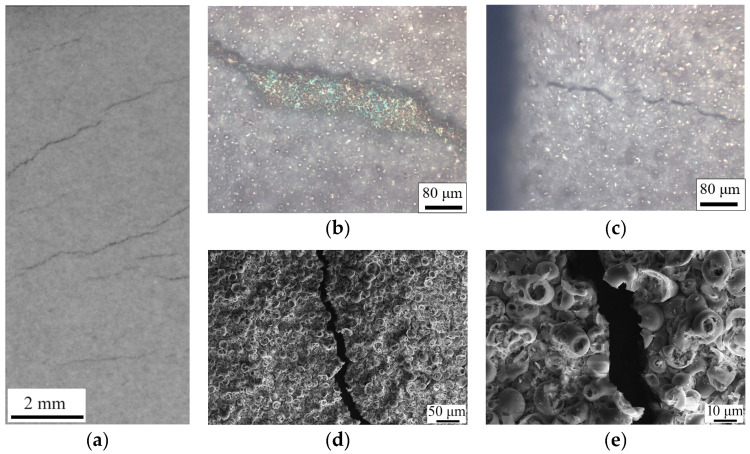
Photograph (**a**), optical images (**b**,**c**), and SEM images (**d**,**e**) of the surface of samples with CaP coatings after tensile test.

The destruction pattern of the CaP coatings, irrespective of their placement in the clamps, was characterized by the formation and propagation of cracks. These cracks originated from the boundaries of the working part of the samples and moved perpendicular to the applied load. As the load increased linearly, the cracks widened, exposing the substrate, and traversed across the center of the sample to the opposite boundary (Figure 6). This behavior mirrors other studies [45], where brittle coatings on ductile substrates also exhibited crack propagation perpendicular to the tensile force.

This phenomenon suggests that the brittle nature of the CaP coating, when paired with a ductile substrate like Ti, may actually limit the overall deformation capability of the composite. Thus, despite the CaP coating’s contribution to the enhancement of mechanical properties in the elastic region, as demonstrated earlier, its inherent brittleness could be a limiting factor in applications requiring higher deformation capacities. These findings highlight the need for a balanced approach in composite design, where the benefits of increased strength and elasticity must be weighed against the potential limitations imposed by the material’s brittleness. Future research should focus on exploring coatings with modified brittleness or alternative composite configurations that could offer an optimal balance between strength, elasticity, and ductility for specific biomedical applications. Given the method of deposition used, it is likely that the high internal and surface porosity, as well as the roughness (*Ra* = 3.5 µm), contribute to the mechanical properties of the CaP coatings. The porosity, while beneficial for drug delivery due to increased surface area, can also introduce stress concentrations that become focal points for crack initiation. In order to study it more directly, SEM imaging was performed (Figure 6d,e). 

The sharpness of the crack edges suggests a brittle fracture, which typically occurs without significant plastic deformation. This is consistent with the low residual deformation (1.3%) noted earlier, indicating that the material failed in a brittle manner. The observation that some spheres within the coating are cracked in half while others remain intact could indicate a variation in local stress distribution or a difference in the structural integrity of individual spheres. This heterogeneity might be due to differences in material density, defects, or bonding strength between the spheres and the matrix.

The exact point of crack initiation is challenging to determine post facto, but it often occurs at sites of stress concentration. These could be defects, such as pores or inclusions, or areas with significant microstructural variations. The uniform appearance of the spheres suggests that material properties are consistent across the surface, yet the crack path indicates selective fracture along specific points of weakness. By comparing the coated sample that was clamped and subsequently failed at a higher stress with the one that was not clamped, we can hypothesize that the clamping could have introduced additional external stress or altered the internal stress distribution, affecting the crack initiation point and the failure mode. The differences in crack initiation and propagation between the spheres that were included within the clamp and those not clamped might indicate differences in adhesion to the substrate. Better adhesion could lead to stress being more effectively transferred to the coating, which could then fail at higher stresses.

### 3.4. Biodegradation of PLGA/CaP/Ti Composites

**Figure 7:**
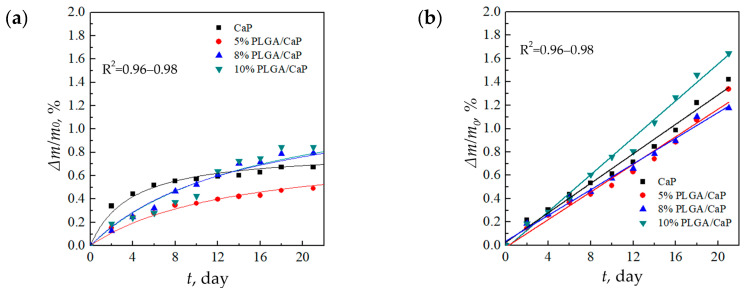
Relative rate of degradation of support matrices in solutions of 0.9% NaCl (**a**) and PBS (**b**).

Gravimetric analysis over a 21 days period revealed a hyperbolic trend in weight loss for all samples when immersed in the 0.9% NaCl solution, as depicted in Figure 7a. Notably, the 5% PLGA/CaP/Ti samples demonstrated the least weight loss, which might be due to smaller mass of the polymer incapsulating layer.

Conversely, in the PBS medium, the samples experienced a consistent linear weight loss over time depicted in Figure 7b. By the end of the 21 days duration, the weight loss in PBS was significantly higher—ranging between 1.1 and 1.7%—compared to 0.3–0.8% in the 0.9% NaCl solution. This delineates PBS as a more corrosive environment for these composites, potentially due to its phosphate content, which may accelerate degradation processes, as shown in Figure 7b.

The relatively modest weight losses, not surpassing 1.7%, are indicative of the localized biodegradation of the composite’s upper layers, which comprise a mere 4 wt.% of the total composition. The remainder of the composite, primarily consisting of the insoluble Ti substrate, remains intact. These observations are in alignment with the literature, where, for example, the weight loss rate of PLGA in Hanks’ solution is reported to be linear, reaching 33% after 36 days [46]. Similarly, a significant weight loss of PLGA in a PBS solution is documented to occur after the initial 2–3 weeks of immersion [47]. The reported results indicate the importance of corrosive media to the corrosion behavior of the composite.

### 3.5. Electrochemical Properties of PLGA/CaP/Ti Composites

**Figure 8:**
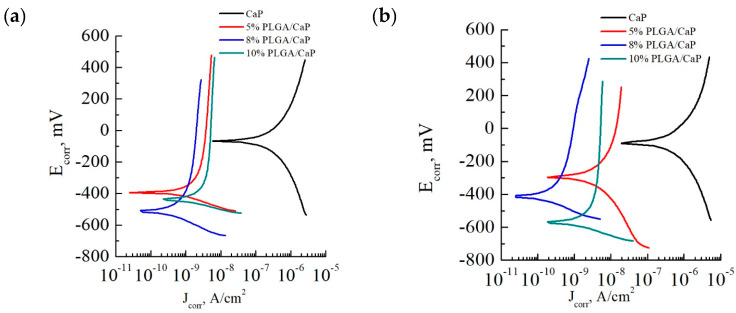
Tafel polarization curves taken for the corresponding sample groups in solutions of 0.9% NaCl (**a**) and PBS (**b**).

In our electrochemical analysis, we utilized the potentiodynamic polarization method to gain insights into the corrosion resistance of various sample groups. Through this method, Tafel polarization curves were plotted and key parameters such as corrosion current, corrosion potential, and polarization resistance were meticulously determined, as illustrated in Figure 8 and documented in Table 2. Tafel extrapolation, derived from these polarization curves, is a technique to assess the corrosion kinetics of materials. It offers valuable information on the electrochemical processes occurring at the material’s surface, which is essential for understanding the protective efficacy of CaP coatings applied to metallic substrates. In examining the Tafel plots, we observed that the CaP/Ti samples exhibited relatively low corrosion currents of 228.2 and 355.0 × 10^−9^ A/cm^2^, alongside substantial polarization resistances of 0.3 and 0.2 × 10^6^ Ω·cm^2^ for 0.9% NaCl and PBS solutions, respectively. Notably, the increment in corrosion current and corresponding decrease in polarization resistance within the PBS solution underscore the solution’s aggressive nature, which accelerates biocorrosion.

For perspective, according to the literature [48], pure Ti exhibits a corrosion current of 10–20 × 10^−6^ A/cm^2^. This comparison highlights the enhanced corrosion resistance afforded by the CaP coating, which is approximately two orders of magnitude greater than that of pure Ti.

The incorporation of a PLGA polymer into the CaP coating further augmented the corrosion resistance. Introducing even a mere 5% PLGA layer resulted in a dramatic reduction in corrosion current—down by two orders of magnitude compared to the CaP/Ti control—to 0.6 and 3.8 × 10^−9^ A/cm^2^ in 0.9% NaCl and PBS solutions, respectively. Similarly, the polarization resistance saw a marked increase to 37.8 and 20.7 × 10^6^ Ω·cm^2^ in the same solutions. These figures emphasize the significant insulating effect provided by the thin PLGA layer, even at minimal application mass.

Elevating the PLGA concentration to 8–10% enhanced the coating’s thickness, which further decreased the corrosion currents to nearly negligible levels. Such results could suggest an almost complete corrosion resistance in the multi-level systems we developed. To contextualize, a PLGA coating on a steel substrate has a reported corrosion current of 8.91 × 10^−6^ A/cm^2^ [49], which is substantially higher—by four orders of magnitude—than our systems. Moreover, other researchers have successfully decreased the corrosion current of magnesium alloys to 0.69 × 10^−6^ A/cm^2^ through surface modification with composite layers of CaP and PLGA nanopowder [50], and even to 3.1 × 10^−9^ A/cm^2^ by applying a dense polymer layer of ultrafine polytetrafluoroethylene [51]. Therefore. our study demonstrates the potential for polymer-based modifications to significantly extend the lifespan of biomedical implants by mitigating corrosion—a pivotal factor for in vivo applications.

**Figure 9:**
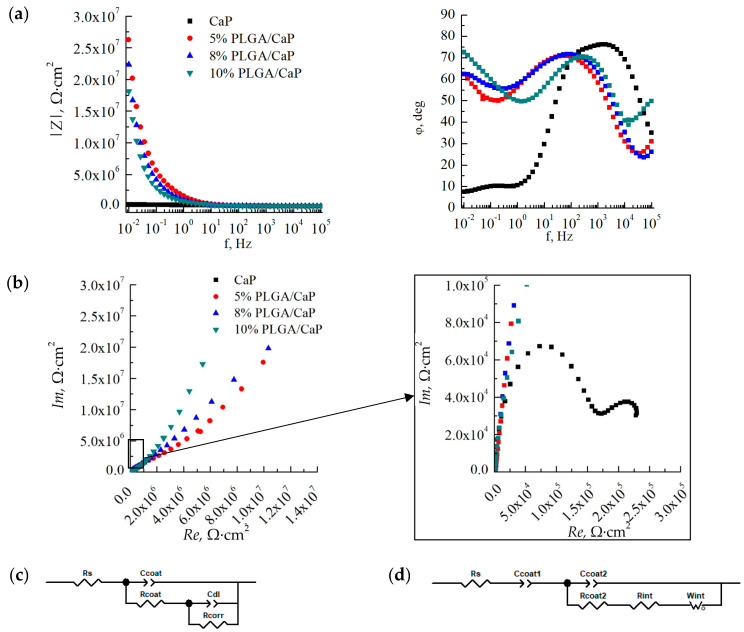
Bode (**a**) and Nyquist (**b**) plots at various scales, based on the results of impedance spectroscopy in a solution of 0.9% NaCl, as well as simulated equivalent electrical circuits for CaP/Ti (**c**) and PLGA/CaP/Ti (**d**) samples.

Through the application of EIS, we have been able to elucidate the electrochemical behavior of corresponding samples in different simulated biological environments—0.9% NaCl and PBS. The impedance spectra we recorded revealed distinct characteristics for the CaP/Ti and PLGA/CaP/Ti, which held true across all concentrations of the polymer (Figure 9). However, we did not notice a significant difference in impedance spectra upon a change in the medium. The character of impedance spectra in PBS medium of CaP/Ti and PLGA/CaP/Ti at different concentrations were found to have insignificant differences.

EIS is used for evaluating the integrity and protective quality of coatings. It allows us to investigate the stability of CaP and PLGA coatings, monitor porosity changes over time, and assess the coatings’ efficacy as barriers against substrate corrosion. Modelled equivalent electrical circuits revealed differences between the CaP/Ti and PLGA/CaP/Ti sample groups. This discrepancy can largely be attributed to the formation of a double electrical layer on the polymer-modified surfaces and the differing electrical capacitances of these layers. For the CaP/Ti, we noted an electrical capacitance around 0.1 μF. In contrast, the PLGA/CaP/Ti systems exhibited such high capacitance at the specified frequency range that it was effectively immeasurable, necessitating the modeling of this component as an open Warburg element (Figure 9c,d).

This behavior was most evident in the 10% PLGA/CaP/Ti variant, which, due to the greater thickness of the polymer layer, displayed a phase shift angle at higher frequencies of the electrical signal when compared to the 5% or 8% PLGA/CaP/Ti systems. However, this characteristic was consistent across all PLGA-modified samples.

By analyzing the impedance spectra, including Bode and Nyquist diagrams, we determined the electrical impedance modulus at a low frequency of 5·10^−2^ Hz. This modulus was found to increase by two orders of magnitude in the PLGA/CaP/Ti system relative to the unmodified CaP coating (Table 3), signifying a substantial boost in corrosion resistance. While the variance in electrical impedance moduli between different PLGA concentrations did not yield statistically significant differences, the trends in circuit constants such as electrical capacitance and resistance suggest a positive correlation between increased PLGA concentration and enhanced corrosion resistance.

Our findings indicate that the PLGA polymer composition plays a pivotal role in reinforcing the barrier properties of the carrier matrices, which is essential for their application in corrosive biological environments. Further studies may delve into the long-term stability of these modifications and explore the implications of electrical behavior on the physical and chemical integrity of the coatings.

## 4. Conclusions

Through two orthogonal adhesion tests, we revealed that the 5% and 8–10% PLGA/CaP/Ti coatings significantly enhance adhesive–cohesive strength when compared to the unmodified CaP coating. Interestingly, we were able to determine PLGA to CaP adhesion using peel testing, and it was revealed that the critical load for PLGA/CaP/Ti samples was found to be not less than 8.1 ± 2.2 MPa. 

The tensile stress–strain behavior of the CaP/Ti composite revealed predominantly brittle fracture in the CaP coatings, with crack initiation points aligning with stress concentrations at the porous inter-spherical boundaries after tensile testing. 

The gravimetric approach indicated a hyperbolic trend in weight loss over 21 days, with the lowest weight loss observed in the 5% PLGA/CaP/Ti sample in 0.9% NaCl medium. In contrast, PBS proved to be a more aggressive medium, doubling the corrosion rate compared to the 0.9% NaCl environment. 

Potentiodynamic polarization studies revealed low corrosion currents of 228.2 and 355.0 × 10^−9^ A/cm^2^ for unmodified CaP in 0.9% NaCl and PBS, respectively. The addition of PLGA reduced the corrosion current even further. Electrochemical impedance spectroscopy underscored the significant insulation effect of the PLGA layer. The 5% PLGA/CaP/Ti variant demonstrated an increase in impedance modulus by two orders of magnitude over the CaP coating. The findings of this study highlight the effectiveness of the PLGA polymer composition in improving both the mechanical and corrosion-resistant properties of CaP coatings even when ultimately thin layers of polymer are deposited, which we showed, as an example, for 5% PLGA/CaP/Ti. The application of PLGA not only enhanced the structural integrity of the coatings under mechanical stress but also substantially mitigated the corrosive processes in simulated biological environments.

## Figures and Tables

**Table 1:** Adhesion strength of the CaP and PLGA/CaP coatings to the Ti substrate in peel adhesion and scratch tests.

Sample Group	Adhesion Strength (Peel Adhesion Test), MPa	Critical Load, H		
		CL1	CL2	CL3
CaP/Ti	20.1 ± 1.6	0.5 ± 0.1	9.4 ± 3.2	30.3 ± 5.2
5% PLGA/CaP/Ti	15.6 ± 5.9	1.2 ± 0.1	15.5 ± 4.0	33.3 ± 4.6
8% PLGA/CaP/Ti	9.8 ± 3.8	1.3 ± 0.1	23.5 ± 0.9	35.0 ± 0.8
10% PLGA/CaP/Ti	8.1 ± 2.2	1.2 ± 0.1	25.6 ± 3.5	36.0 ± 1.9

**Table 2:** Corrosion characteristics of carrier matrix options in solutions of 0.9% NaCl and PBS.

Sample Group	E_ocp_, V	E_corr_, V	I_corr_, ×10^−9^ A/cm^2^	R_p_, ×10^6^ Ω·cm^2^
0.9% NaCl				
CaP/Ti	−0.044	−0.065	228.2	0.3
5% PLGA/CaP/Ti	−0.051	−0.393	0.6	37.8
8% PLGA/CaP/Ti	−0.061	−0.508	0.3	91.1
10% PLGA/CaP/Ti	−0.068	−0.433	0.6	24.1
PBS				
CaP/Ti	−0.029	−0.088	355.0	0.2
5% PLGA/CaP/Ti	−0.030	−0.345	3.8	20.7
8% PLGA/CaP/Ti	−0.061	−0.566	1.1	17.8
10% PLGA/CaP/Ti	−0.057	−0.416	0.1	26.7

Note: E_ocp_ is an open circuit potential; E_corr_ is a corrosion potential; I_corr_ is a corrosion current density; R_p_ is a corrosion resistance.

**Table 3:** Electrical impedance of carrier matrices in solutions of 0.9% NaCl and PBS, as well as parameters of the equivalent electrical circuit responsible for the properties of the coating.

Sample Group	|Z|, ×10^6^ Ω·cm^2^	C_coat_, F·cm^−2^	R_coat_, ×10^6^ Ω·cm^2^
0.9% NaCl			
CaP/Ti	0.2	7 × 10^−8^	9 × 10^4^
5% PLGA/CaP/Ti	26.3	4 × 10^−8^	9 × 10^4^
8% PLGA/CaP/Ti	22.4	5 × 10^−8^	9 × 10^4^
10% PLGA/CaP/Ti	18.1	3 × 10^−8^	9 × 10^4^
PBS			
CaP/Ti	0.1	1.2 × 10^−7^	8 × 10^−2^
5% PLGA/CaP/Ti	1.3	3.4 × 10^−7^	1
8% PLGA/CaP/Ti	15.0	1 × 10^−8^	9 × 10^4^
10% PLGA/CaP/Ti	19.6	5 × 10^−8^	9 × 10^4^

Note: |Z| is a total electrical resistance of the circuit (impedance); C_coat_ is an electrical capacitance of the circuit element responsible for the CaP coating; R_coat_ is a resistance of the circuit element.

## Data Availability

The raw data supporting the conclusions of this article will be made available by the authors on request.
